# Independent evaluation of 12 artificial intelligence solutions for the detection of tuberculosis

**DOI:** 10.1038/s41598-021-03265-0

**Published:** 2021-12-13

**Authors:** Andrew J. Codlin, Thang Phuoc Dao, Luan Nguyen Quang Vo, Rachel J. Forse, Vinh Van Truong, Ha Minh Dang, Lan Huu Nguyen, Hoa Binh Nguyen, Nhung Viet Nguyen, Kristi Sidney-Annerstedt, Bertie Squire, Knut Lönnroth, Maxine Caws

**Affiliations:** 1 Friends for International TB Relief (FIT), Ho Chi Minh City, Viet Nam; 2 IRD VN, Ho Chi Minh City, Viet Nam; 3 Department of Global Public Health, WHO Collaboration Centre on Tuberculosis and Social Medicine, Karolinska Institutet, Solna, Sweden; 4 Pham Ngoc Thach Hospital, Ho Chi Minh City, Viet Nam; 5 National Lung Hospital, Ha Noi, Viet Nam; 6 Department of Clinical Sciences, Liverpool School of Tropical Medicine (LSTM), Liverpool, UK; 7 Birat Nepal Medical Trust, Kathmandu, Nepal

**Keywords:** Epidemiology, Machine learning

## Abstract

There have been few independent evaluations of computer-aided detection (CAD) software for tuberculosis (TB) screening, despite the rapidly expanding array of available CAD solutions. We developed a test library of chest X-ray (CXR) images which was blindly re-read by two TB clinicians with different levels of experience and then processed by 12 CAD software solutions. Using Xpert MTB/RIF results as the reference standard, we compared the performance characteristics of each CAD software against both an Expert and Intermediate Reader, using cut-off thresholds which were selected to match the sensitivity of each human reader. Six CAD systems performed on par with the Expert Reader (Qure.ai, DeepTek, Delft Imaging, JF Healthcare, OXIPIT, and Lunit) and one additional software (Infervision) performed on par with the Intermediate Reader only. Qure.ai, Delft Imaging and Lunit were the only software to perform significantly better than the Intermediate Reader. The majority of these CAD software showed significantly lower performance among participants with a past history of TB. The radiography equipment used to capture the CXR image was also shown to affect performance for some CAD software. TB program implementers now have a wide selection of quality CAD software solutions to utilize in their CXR screening initiatives.

## Introduction

An estimated 10 million people developed tuberculosis (TB) globally in 2019, yet just 71% of these individuals were reported as treated^1^. The persistent gap between TB treatment and incidence is a major barrier to achieving TB elimination^2^. Numerous intensified and active TB case finding approaches have been piloted over the past decade to identify and treat people with TB who are missed by existing health services^3^. Chest X-ray (CXR) is known to be one of the most sensitive screening tools for TB, but its widespread application in high TB burden countries has traditionally been limited, in line with past World Health Organization policy advising against TB case finding by mobile mass radiography^4^. In recent years, there has been a renewed interest in CXR screening for TB due advances in digital radiography equipment and the advent molecular diagnostic assays^5^.

Several large-scale, community-based CXR screening initiatives have been recently implemented in high TB burden countries^6–11^. These programs show that this case finding approach is both feasible to implement in low- and middle-income countries (LMIC) and effective at identifying people with TB, particularly those with subclinical disease who are frequently missed by TB programs or only diagnosed after long delays^12^. However, most LMIC health systems do not have sufficient capacity to systematically implement this approach. A recent survey of National TB Programs (NTPs) and local implementers from 22 high TB burden countries indicated that 59% were concerned about shortages of qualified radiologists when planning their own CXR screening initiatives^13^. In addition, high levels of inter- and intra-reader variability can make ensuring the quality of CXR image interpretation a real challenge during programmatic screening activities.

Artificial Intelligence (AI) is one of the fastest growing fields of technology^14^, and it is increasingly being applied to diverse challenges in healthcare, including drug discovery^15,16^, healthcare management^17^, and disease diagnosis^18^. AI also has significant potential to improve TB screening^19^. Multiple computer-aided detection (CAD) software solutions have been developed which can systematically assess and interpret CXR images in the absence of a radiologist. CAD software for TB screening produce a continuous abnormality score which indicates the likelihood that a CXR image contains an abnormality associated with TB. These scores can then be dichotomized at a selected threshold, above which the CXR image is categorized as abnormal and the individual is indicated for further TB evaluations, such as a sputum-based molecular diagnostic test. The AI algorithms in some CAD solutions will automatically select a cut-off threshold for users, and will continuously use follow-on sputum test result data to optimize threshold selection.

The majority of the published literature on CAD software for TB screening has focused on Delft Imaging’s CAD4TB (The Netherlands), which was one of the first commercially available CAD solutions^8,11,20–23^. Two systematic reviews, conducted in 2016 and 2019, also primarily included studies evaluating various versions of the CAD4TB software^24,25^. More recent evaluations have included additional CAD software solutions, including Qure.ai’s qXR (India), Lunit’s INSIGHT CXR (South Korea), JF Healthcare’s JF CXR-1 (China) and InferVision’s InferRead DR Chest (Japan)^26–29^. These early evaluations suggest that CAD solutions can match the performance of experienced human readers for detecting abnormalities associated with TB. However, there have been limited reports of independent evaluations applying the technology under programmatic conditions. Continuous software version updates have further complicated the systematic evaluation of different CAD software solutions.

We developed a well-characterized test library of CXR images derived from a community-based, mobile CXR screening initiative in Viet Nam^9^, and then identified and approached CAD companies for participation in an independent, comparative evaluation of their newest CAD software versions.

## Results

### CXR test library characteristics

Of the 1032 participants included in the final test library, 133 (12.9%) had a positive Xpert result (Table 1). The test library contains more male than female participants (69.0% vs 31.0%) and Xpert positivity is significantly higher in males (15.0% vs 8.1%, p = 0.002), consistent with the TB epidemiology in the source population^30^. The test library also contains a higher proportion participants aged ≥ 55 years (71.8% vs 28.2%), yet Xpert positivity is significantly higher in the younger cohort (11.1% vs 17.5%, p = 0.005). Only 39.0% of test library participants reported having a cough lasting two weeks or longer (a common screening criteria for indicating TB diagnostic evaluations in Viet Nam). 38.2% of test library participants reported having no cough, fever, weight loss or night sweats. Approximately a third of test library participants (33.5%) reported having an episode of TB in the past; however, the proportion who were Xpert positive was not significantly different between those with and without a prior episode of TB (15.0% vs 12.1%, p = 0.145). Approximately half of the CXR images were captured by each of the library’s radiography systems: JPI Healthcare and DRTECH (47.8% and 52.8%, respectively). Xpert positivity was significantly higher among participants screened with the DRTECH radiography system (23.9% vs 6.3%, p < 0.001). The Expert Reader classified 62.7% of the images in the test library as Abnormal, while the Intermediate Reader classified 48.0% of the images as Abnormal. The Intermediate Reader’s classifications would have resulted in 24 Xpert positive participants being classified as Normal and not being indicated for further TB testing. The Expert Reader only classified the CXR images of six Xpert positive participants as Normal (4.5% vs 1.6%, p = 0.014).

**Table 1:** Demographic and clinical description of participants included in the test library.

	Total (N, %)	Xpert MTB/RIF results		P-value^╪^
		Negative (N, %)	Positive (N, %)	
All participants	1032	899 (87.1%)	133 (12.9%)	N/A
**Demographic factors**				
Gender				
Male	712 (69.0%)	605 (85.0%)	107 (15.0%)	**0.002**
Female	320 (31.0%)	294 (91.9%)	26 (8.1%)	
Age, median (IQR)	62 (53–70)	62 (54–71)	59 (48–68)	**0.004**
15–54 years	291 (28.2%)	240 (82.5%)	51 (17.5%)	**0.005**
≥ 55 years	741 (71.8%)	659 (88.9%)	82 (11.1%)	
Health insurance	881 (85.4%)	769 (87.3%)	112 (12.7%)	0.686
Residency status				
Long-term resident of HCMC	896 (86.8%)	783 (87.4%)	113 (12.6%)	0.497
Recent migrant to HCMC	136 (13.2%)	116 (85.3%)	20 (14.7%)	
**Presence of TB symptoms**				
Cough (C)				
No Cough	455 (44.1%)	420 (92.3%)	35 (7.7%)	** < 0.001**
< 2 weeks	175 (17.0%)	148 (84.6%)	27 (15.4%)	
≥ 2 weeks	402 (39.0%)	331 (82.3%)	71 (17.7%)	
Fever (F)	56 (5.4%)	43 (76.8%)	13 (23.2%)	**0.018**
Weight loss (WL)	113 (10.9%)	94 (83.2%)	19 (16.8%)	0.187
Night sweats (NS)	64 (6.2%)	59 (92.2%)	5 (7.8%)	0.211
4 Symptoms: C + F + WL + NS	638 (61.8%)	534 (83.7%)	104 (16.3%)	** < 0.001**
Chest pain	229 (22.2%)	199 (86.9%)	30 (13.1%)	0.913
Fatigue	235 (22.8%)	189 (80.4%)	46 (19.6%)	** < 0.001**
Any TB symptom	727 (70.4%)	614 (84.5%)	113 (15.5%)	** < 0.001**
Cough plus any one symptom	291 (28.2%)	238 (81.8%)	53 (18.2%)	**0.001**
Any TB symptom except cough	441 (42.7%)	373 (84.6%)	68 (15.4%)	**0.036**
**TB risk factors**				
Contact of TB patient	64 (6.2%)	48 (75.0%)	16 (25.0%)	**0.003**
Past history of TB	346 (33.5%)	294 (85.0%)	52 (15.0%)	0.145
Diabetes	103 (10.0%)	87 (84.5%)	16 (15.5%)	0.398
HIV	2 (0.2%)	2 (100.0%)	0 (0.0%)	0.586
**X-ray factors**				
Radiography system				
JPI Healthcare	493 (47.8%)	464 (94.1%)	29 (6.3%)	** < 0.001**
DRTECH	539 (52.2%)	435 (80.7%)	104 (23.9%)	
Abnormal CXR				
Expert Reader	647 (62.7%)	520 (80.4%)	127 (19.6%)	0.338
Intermediate Reader	495 (48.0%)	386 (78.0%)	109 (22.0%)	
Normal/Clear CXR				
Expert Reader	385 (37.3%)	379 (98.4%)	6 (1.6%)	**0.014**
Intermediate Reader	537 (52.0%)	513 (95.5%)	24 (4.5%)	

Significant values are in bold.

^╪^Chi-squared test.

### CAD software performance

**Figure 1:**
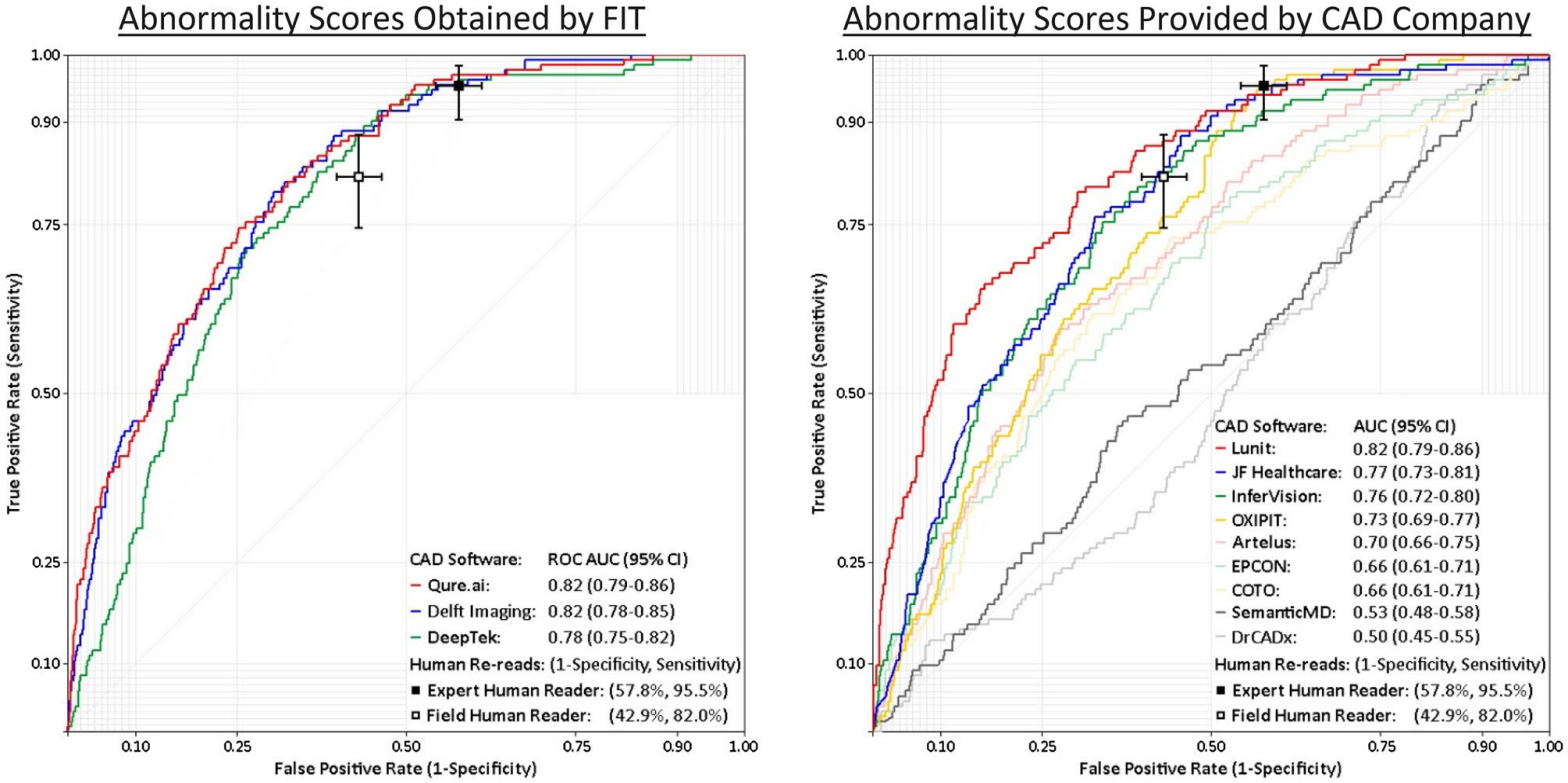
ROC graphs for each CAD software. *ROC AUC* area under the receiver operating characteristic curve.

Table 2 shows the receiver operating characteristic (ROC) area under the curve (AUC) and precision-recall (PR) AUC for each CAD software and Fig. 1 shows their respective ROC curves. Both Qure.ai’s qXR v3 and Delft Imaging’s CAD4TB v7 achieved a ROC AUC of 0.82, and both software had similar PR AUCs (0.41 for Qure.ai and 0.39 for Delft Imaging). DeepTek’s Genki v2 (India) achieved a ROC AUC of 0.78 (0.75–0.82), which is non-significantly lower than the ROC AUC of qXR v3 and CAD4TB v7. Among the software which were evaluated after the provision of outputs by the software developpers, Lunit’s INSIGHT CXR v3.1.0.0 was the strongest performer, with a ROC AUC of 0.82 and a PR AUC of 0.44. The ROC AUC of JF Healthcare’s JF CXR-1 v3.0 and InferVision’s InferRead DR Chest v1.0.0.0 were non-significantly lower than the ROC AUC of Lunit. The ROC AUC values for the remaining six CAD software ranged from 0.73 to 0.50.

**Table 2:** Area under the ROC and precision-recall (RC) curves for each CAD software.

Developer (software name^╪^, version)	ROC AUC (95% CI)	PR AUC (95% CI)
**Abnormality scores obtained by FIT**		
Qure.ai (qXR v3)	0.82 (0.79–0.86)	0.41 (0.33–0.50)
Delft Imaging (CAD4TB v7)	0.82 (0.78–0.85)	0.39 (0.31–0.47)
DeepTek (Genki v2)	0.78 (0.75–0.82)	0.28 (0.22–0.34)
**Abnormality scores provided by CAD company**		
Lunit (INSIGHT CXR v3.1.0.0)	0.82 (0.79–0.86)	0.44 (0.35–0.54)
JF Healthcare (JF CXR-1 v3.0)	0.77 (0.73–0.81)	0.28 (0.22–0.35)
InferVision (InferRead DR Chest v1.0.0.0)	0.76 (0.72–0.80)	0.29 (0.22–0.36)
OXIPIT (ChestEye v1)	0.73 (0.69–0.77)	0.23 (0.18–0.28)
Artelus (T-Xnet v1)	0.70 (0.66–0.74)	0.23 (0.17–0.29)
EPCON (XrayAME v1)	0.66 (0.61–0.71)	0.23 (0.17–0.28)
COTO (v1)	0.66 (0.61–0.71)	0.22 (0.17–0.28)
SemanticMD (v1)	0.53 (0.48–0.58)	0.14 (0.10–0.17)
Dr CADx (v0.1)	0.50 (0.45–0.55)	0.13 (0.10–0.16)

*ROC AUC* area under the receiver operating characteristic curve, *PR AUC* area under the precision-recall curve.

^╪^Software name omitted if none available.

### Comparison of CAD software and human readers

The Expert Reader achieved a sensitivity of 95.5%, a specificity of 42.2% and an accuracy of 49.0% (Table 3). When the cut-off threshold for each CAD software was selected to match the 95.5% sensitivity of the Expert Reader, no CAD software achieved a significantly higher specificity or accuracy. However, Qure.ai’s specificity was very close to being significantly higher (Qure.ai: 48.7% [45.4–52.0%] vs Expert Reader: 42.2% [38.9–45.5%]). Delft Imaging and DeepTek achieved specificity point estimates which were marginally higher than the Expert Reader, while JF Healthcare, OXIPIT and Lunit had specificity point estimates which were marginally lower than the Expert Reader, but these differences were not significant. The six remaining software in the evaluation had a specificity which was significantly lower than the Expert Reader. Despite achieving a lower ROC AUC than InferVision, the specificity of the OXIPIT software was on par with the Expert Reader due to the distribution of the abnormality scores (visible in steep slope change in the ROC curve, Fig. 1).

**Table 3:** CAD software performance when matching the sensitivity of the Expert Reader.

	Cut-off Score	TP	FP	FN	TN	Sensitivity (95% CI)	Specificity (95% CI)	Accuracy (95% CI)
Expert Reader	N/A	127	520	6	379	95.5% (90.4–98.3)	42.2% (38.9–45.5)	49.0% (45.9–52.1)
**Abnormality scores obtained by FIT**								
Qure.ai	44.1	127	461	6	438	95.5% (90.4–98.3)	48.7% (45.4–52.0)	54.7% (51.7–57.8)
DeepTek	31.1	127	483	6	416	95.5% (90.4–98.3)	46.3% (43.0–49.6)	52.6% (49.5–55.7)
Delft imaging	46.7	127	492	6	407	95.5% (90.4–98.3)	45.3% (42.0–48.6)	51.7% (48.7–54.8)
**Abnormality scores provided by CAD company**								
JF Healthcare	83.4	127	530	6	369	95.5% (90.4–98.3)	41.0% (37.8–44.3)	48.1% (45.0–51.2)
OXIPIT	15.4	127	532	6	367	95.5% (90.4–98.3)	40.8% (37.6–44.1)	47.9% (44.8–51.0)
Lunit	3.0	127	551	6	348	95.5% (90.4–98.3)	38.7% (35.5–42.0)	46.0% (43.0–49.1)
InferVision	53.8	127	661	6	238	95.5% (90.4–98.3)	26.5% (23.6–29.5)	35.4% (32.5–38.4)
Artelus	1.2	127	691	6	208	95.5% (90.4–98.3)	23.1% (20.4–26.0)	32.5% (29.6–35.4)
Dr CADx	27.8	127	790	6	109	95.5% (90.4–98.3)	12.1% (10.1–14.4)	22.9% (20.3–25.6)
SemanticMD	0.4	127	808	6	91	95.5% (90.4–98.3)	10.1% (7.2–10.8)	21.1% (16.6–21.2)
EPCON	0.6	127	815	6	84	95.5% (90.4–98.3)	9.3% (6.6–10.0)	20.4% (16.0–20.6)
COTO	1.5	127	842	6	57	95.5% (90.4–98.3)	6.3% (4.8–8.1)	17.8% (15.5–20.3)

*TP* true positive, *FP* false positive, *FN* false negative, *TN* true negative.

The Intermediate Reader achieved a sensitivity of 82.0%, a specificity of 57.1% and an accuracy of 60.3% (Table 4). When the cut-off threshold was fixed to match the 82.0% sensitivity achieved by the Intermediate Reader, Qure.ai, Delft Imaging and Lunit achieved a significantly higher specificity and accuracy. DeepTek and JF Healthcare achieved a specificity point estimate which was marginally higher than the Intermediate Reader, while the specificity of InferVision and OXIPIT was slightly lower than the Intermediate Reader. The five remaining software solutions had a specificity which was significantly lower than the Intermediate Reader.

**Table 4:** CAD software performance when matching the sensitivity of the Intermediate Reader.

	Cut-off Score	TP	FP	FN	TN	Sensitivity (95% CI)	Specificity (95% CI)	Accuracy (95% CI)
Intermediate Reader	N/A	109	386	24	513	82.0% (74.4–88.1)	57.1% (53.8–60.3)	60.3% (57.2–63.3)
**Abnormality scores obtained by FIT**								
Qure.ai	76.5	109	307	24	592	82.0% (74.4–88.1)	**65.9% (62.7–69.0)**	**67.9% (65.0–70.8)**
Delft Imaging	64.7	109	309	24	590	82.0% (74.4–88.1)	**65.6% (62.4–68.7)**	**67.7% (64.8–70.6)**
DeepTek	55.7	109	331	24	568	82.0% (74.4–88.1)	63.2% (59.9–66.3)	65.6% (62.6–68.5)
**Abnormality scores provided by CAD company**								
Lunit	20.7	109	314	24	585	82.0% (74.4–88.1)	**65.1% (61.9–68.2)**	**67.2% (64.3–70.1)**
JF Healthcare	98.3	109	379	24	520	82.0% (74.4–88.1)	57.8% (54.5–61.1)	60.9% (57.9–63.9)
InferVision	77.4	109	387	24	512	82.0% (74.4–88.1)	57.0% (53.6–60.2)	60.2% (57.1–63.2)
OXIPIT	23.8	109	441	24	458	82.0% (74.4–88.1)	50.9% (47.6–54.3)	54.9% (51.9–58.0)
Artelus	5.6	109	492	24	431	82.0% (74.4–88.1)	45.3% (42.0–48.6)	50.0% (46.9–53.1)
EPCON	11.7	109	547	24	352	82.0% (74.4–88.1)	39.2% (36.0–42.4)	44.7% (41.6–47.8)
COTO	12.2	109	568	24	331	82.0% (74.4–88.1)	36.8% (33.7–40.1)	42.6% (39.6–45.7)
Dr CADx	64.1	108	713	25	186	81.2% (73.5–87.5)^╪^	20.7% (18.1–23.5)	28.5% (25.8–31.4)
SemanticMD	0.9	109	714	24	185	82.0% (74.4–88.1)	20.6% (18.0–23.4)	28.5% (25.8–31.4)

*TP* true positive, *FP* false positive, *FN* false negative; *TN* True Negative.

Bolded figures indicate performance significantly higher than the Intermediate Reader.

^╪^It was impossible to select a cut-off score achieving 109 true positives, as two Xpert-positive participants have the same score.

### Factors affecting CAD software performance

Table 5 shows the ROC AUC for the top seven performing CAD software (performance at least on par with the Intermediate Reader) disaggregated by key demographic and clinical factors. No software recorded significant differences in ROC AUC between male and female participants; however, the ROC AUC difference by sex of JF Healthcare (0.74 vs 0.82, p = 0.057) and DeepTek (0.75 vs 0.83, p = 0.066) approached statistical significance. The Delft Imaging (0.86 vs 0.79, p = 0.48) and JF Healthcare (0.82 vs 0.74, p = 0.044) software solutions showed significant differences in ROC AUC between younger (15–54 years) and older (≥ 55 years) participants. All seven of the top performing CAD software solutions showed no significant differences between test library participants with and without TB symptoms. However, all but one software (InferVision) showed highly significant differences in ROC AUC between test library participants who reported a history of TB and those who did not. The largest differences between these two cohorts were recorded by JF Healthcare (0.82 vs 0.66, p < 0.001) and OXIPIT (0.79 vs 0.63, p < 0.001). Lastly, the ROC AUC for the OXIPIT (0.77 vs 0.65, p = 0.006) and DeepTek (0.81 vs 0.72, p = 0.027) software varied significantly depending on the radiography system used to capture the CXR image, while there was weak statistical evidence that the differences observed for the Delft Imaging software were not due to random chance (0.82 vs 0.79, p = 0.514).

**Table 5:** Comparison of CAD software ROC AUC by key demographic and clinical factors.

	Qure.ai		Delft Imaging		DeepTek			
	ROC AUC (95% CI)	P-value^╪^	ROC AUC (95% CI)	P-value^╪^	ROC AUC (95% CI)	P-value^╪^		
	0.82 (0.79–0.85)	–	0.82 (0.78–0.85)	–	0.78 (0.74–0.82)	–		
**Gender**								
Male	0.80 (0.76–0.85)	0.222	0.80 (0.76–0.85)	0.363	0.75 (0.71–0.80)	0.066		
Female	0.85 (0.79–0.92)		0.84 (0.78–0.90)		0.83 (0.76–0.90)			
**Age group**								
15–54 years	0.84 (0.79–0.90)	0.248	0.86 (0.81–0.91)	**0.048**	0.79 (0.73–0.85)	0.534		
≥ 55 years	0.80 (0.76–0.85)		0.79 (0.74–0.84)		0.77 (0.72–0.82)			
**C + F + WL + NS**								
No	0.78 (0.71–0.86)	0.294	0.78 (0.72–0.85)	0.307	0.75 (0.67–0.83)	0.489		
Yes	0.83 (0.79–0.87)		0.82 (0.78–0.86)		0.78 (0.74–0.82)			
**History of TB**								
No	0.86 (0.83–0.90)	**0.002**	0.85 (0.82–0.89)	**0.019**	0.83 (0.79–0.87)	**0.001**		
Yes	0.73 (0.65–0.80)		0.76 (0.69–0.83)		0.69 (0.62–0.77)			
**Radiography system**								
JPI Healthcare	0.85 (0.79–0.90)	0.072	0.82 (0.76–0.87)	0.514	0.81 (0.75–0.86)	**0.027**		
DRTECH	0.78 (0.73–0.83)		0.79 (0.75–0.84)		0.72 (0.67–0.77)			

	Lunit		JF Healthcare		InferVision		OXIPIT	
	ROC AUC (95% CI)	P-value^╪^	ROC AUC (95% CI)	P-value^╪^	ROC AUC (95% CI)	P-Value^╪^	ROC AUC (95% CI)	P-value^╪^
	0.82 (0.79–0.86)	–	0.77 (0.73–0.81)	–	0.76 (0.72–0.80)	–	0.73 (0.69–0.77)	–
**Gender**								
Male	0.81 (0.77–0.86)	0.373	0.74 (0.69–0.79)	0.057	0.75 (0.70–0.79)	0.712	0.70 (0.66–0.75)	0.718
Female	0.85 (0.79–0.90)		0.82 (0.75–0.90)		0.77 (0.68–0.86)		0.77 (0.69–0.84)	
**Age group**								
15–54 years	0.83 (0.77–0.89)	0.771	0.82 (0.76–0.87)	**0.044**	0.79 (0.73–0.85)	0.127	0.75 (0.69–0.81)	0.335
≥ 55 years	0.82 (0.77–0.86)		0.74 (0.69–0.79)		0.73 (0.67–0.78)		0.71 (0.66–0.76)	
**C + F + WL + NS**								
No	0.79 (0.72–0.85)	0.244	0.71 (0.63–0.80)	0.142	0.73 (0.64–0.82)	0.525	0.72 (0.64–0.80)	0.911
Yes	0.83 (0.79–0.88)		0.78 (0.74–0.83)		0.76 (0.72–0.81)		0.72 (0.68–0.77)	
**History of TB**								
No	0.86 (0.82–0.90)	**0.019**	0.82 (0.77–0.86)	**0.001**	0.78 (0.73–0.83)	0.122	0.79 (0.74–0.83)	** < 0.001**
Yes	0.75 (0.68–0.83)		0.66 (0.59–0.74)		0.71 (0.64–0.78)		0.63 (0.55–0.70)	
**Radiography system**								
JPI Healthcare	0.79 (0.71–0.86)	0.790	0.76 (0.69–0.83)	0.791	0.78 (0.70–0.86)	0.118	0.77 (0.71–0.84)	**0.006**
DRTECH	0.80 (0.75–0.84)		0.75 (0.70–0.80)		0.70 (0.65–0.75)		0.65 (0.60–0.71)	

*ROC AUC* area under the receiver operating characteristic curve.

Significant values are in bold.

^╪^Chi-squared test.

## Discussion

Three CAD software solutions emerged from this evaluation as excellent alternatives for human CXR interpretation, performing on par with the Expert Reader and significantly better than the Intermediate Reader: Qure.ai qXR v3, Delft Imaging CAD4TB v7 and Lunit INSIGHT CXR v3.1.0.0. DeepTek Genki v2 also performed on a par with Expert and Intermediate Readers. Three additional CAD software solutions performed at least on par with the Intermediate Reader.

This evaluation assessed the performance of 12 CAD software solutions for TB screening, which is the largest cross-platform comparative evaluation published to date. This is also the first time six of these CAD solutions have been independently evaluated in the literature. Previous systematic reviews have focused solely on Delft Imaging’s CAD4TB^24,25^, and more recent comparative evaluations^26,27,29^ have included only a limited number of CAD solutions. This independent evaluation highlights the recent significant advances in diagnostic accuracy of multiple CAD software platforms and also identifies important limitations of the CAD software, which should be addressed in future implementation research.

All seven of these top performing CAD software solutions showed equivalent performance among participants with and without TB symptoms. This finding has important implications for the potential of CAD technology to increase the effectiveness of TB screening programs in identifying people with TB, because approximately half of people with active TB disease in the community do not report having TB symptoms: 30–60% of people with TB in Africa^31^ and 40–79% of people with TB in Asia^32^. These individuals can often only be detected through CXR screening, either through community-based screening initiatives or supported by other community referral programs which succeed in overcoming access barriers for facility-based X-ray services^33,34^. CAD software solutions have the potential to reduce CXR access barriers related to shortages of radiologists, particularly those with specialist training in TB.

However, there are key factors which may significantly impair the performance of CAD solutions. Specifically, all but one of the seven top performing CAD software solutions (InferVision) had a significantly lower ROC AUC in people with a history of TB. Participants who had TB in the past may have abnormalities on their CXR images (e.g. fibrotic scarring, nodules without calcification, etc.) which are not indicative of current TB disease. In these instances, a high CAD software abnormality score may be paired with a negative Xpert test, resulting in diminished software performance. In addition, Xpert testing among people with a history of TB can produce false positive Xpert results many months after a patient has successfully completed treatment^35^. Implementers should be aware of this common limitation when integrating CAD software into their TB programs. CXR images from people with a past history of TB may need an alternative threshold or to be reviewed by an experienced human reader. Software companies should develop, evaluate and refine alternative algorithms for this patient group to optimize software performance.

Although all of the seven top performing CAD software solutions indicated they were radiography system agnostic, we observed a significant impairment in the performance of two solutions (OXIPIT and DeepTek) and possibly a third (Delft Imaging) depending on the radiography system used for CXR image capture. However, the test library evaluated contains only two types of radiography systems, and therefore our data suggests broader independent evaluation of all software solutions against a range of radiography equipment is necessary. Many health systems in high TB burden countries have older and poorly maintained radiography equipment in current use.

The high level of inter-reader variability of CXR images has been well documented in TB programs since the late 1960s^36^, particularly among less experienced readers^37^. A strength of this CAD software evaluation was the involvement of two TB clinicians with different levels of experience as benchmarks for the software solutions. This particularly pertains to the inclusion of the Intermediate Reader, as many CAD software evaluations have used a single highly skilled radiologist to re-read the CXR images, thereby setting a very high standard for CAD software diagnostic accuracy^23,27,29^. However, experienced expert TB clinicians and radiologists are unlikely to participate in programmatic CXR screening initiatives on a regular basis. The Expert Reader achieved a 95.5% sensitivity, compared to an 82.0% sensitivity for the Intermediate Reader. The level of experience of this evaluation’s Intermediate Reader is more representative of the field radiologists which Friends for International TB Relief (FIT) employs during mobile CXR screening initiatives. However, the Intermediate Reader is a staff member of a tertiary respiratory hospital, and may be more experienced than generalist radiologist or TB clinicians working at lower-volume secondary and primary care facilities. It is therefore possible that many of the software evaluated in this study would exceed the performance of standard programmatic screening staff, and further evaluations should determine the potential gains in accuracy of screening programs applying CAD solutions.

Now that several CAD software have achieved accuracy exceeding that of human readers, it is also essential to conduct cost effectiveness studies. Our literature review did not find fixed price points published for the CAD software solutions included in this evaluation. Informal feedback from early CAD software adopters has indicated that a unit cost model for each processed DICOM file is commonplace. However, CAD developers may orient themselves on other viable, commonly observed pricing models for SaaS (Software-as-a-Service) solutions, such as per-user subscriptions or price segmentation by time, feature or disease^38,39^. Hybrid pricing models, such as freemium or free/ad-supported solutions, are additional marketing options CAD software developers could consider in light of the increasingly competitive environment of this rapidly expanding market. Lastly, structuring and presenting the chosen pricing model as either value-based or cost-based pricing may also be critical in markets where high-quality and relatively low-cost radiologists are readily available.

Justifying the costs of CAD solutions will most certainly depend on the added value for each individual use case. The FIT mobile CXR screening initiative mobilizes and processes 300 participants per day on average^9^, and one radiologist interprets all of the CXR images in real-time as they are captured throughout the day. In such a high volume setting, CXR interpretation quality and reader fatigue are real concerns^40^. CAD software could be integrated into a screening initiative as an external quality assessment (EQA) tool to identify CXR abnormalities which were missed by the radiologist, or excessive over-reading. Alternatively, the CAD software could be used as a triage tool to identify the totally normal/clear CXR images, reducing the workload of the radiologists and allowing them to prioritize time for reading CXR images which have a higher likelihood of being abnormal. CAD solutions are currently being integrated into mammography screening programs in high-income countries in a similar fashion^41,42^. Further studies evaluating the implementation experiences, software usability and performance of CAD software solutions in these two contexts are urgently needed, particularly for software where diagnostic performance is already well established.

Our study has several limitations. The test library used in this evaluation contains CXR images collected in one region only. CAD software performance may differ across settings and even between the key populations being screened within a setting. The test library was retrospectively constituted using data from the FIT programmatic mobile CXR screening initiatives, and thus it is biased towards persons with suspected TB. It is likely that the CAD software solutions and human readers would correctly identify true negative CXR images with high accuracy. If this cohort of participants was better represented in the test library, the ROC AUC scores for each CAD software and the specificity for human readers and dichotomized CAD software scores would likely be higher. To overcome this limitation, we identified cut-off thresholds that allowed for a direct comparison of CAD software solutions with human readers, who faced the same challenges associated with the test library’s sampling method. We then calculated and compared specificity for the human readers and dichotomized CAD software outputs using Xpert test results as the reference standard for both (primary outcome metric) to minimize the influence of a sampling bias.

A second limitation is that the FIT mobile CXR screening program primarily collected single, spot sputum specimens from participants for Xpert testing. Systematic reviews indicate that the Xpert test has a 99% sensitivity among smear-positive individuals and an 88% sensitivity among smear-negative individuals^43^. However, some systematic TB screening initiatives which have used culture as the gold standard have documented Xpert sensitivity as low as 57%^44^. These data indicate that some test library participants likely have a false negative Xpert result, potentially underestimating CAD software performance. Future CAD evaluations should aim to use the higher sensitivity Xpert MTB/RIF Ultra assay and/or a composite reference standard which includes clinically diagnosed TB after an Xpert-negative result. We were unable to use a composite reference standard in this test library because not all eligible participants underwent a systematic clinical evaluation due to the event-based nature of these campaigns. This evaluation mitigated the impact of unquantified under-diagnosis of TB by focusing on the comparison between human readers and dichotomized CAD software outputs as the primary outcome metric, where performance of both human readers and CAD software were equally affected by the under-diagnosis of TB.

This evaluation collected CAD software outputs using two methods: direct collection by FIT staff who had access to online or box versions of the CAD software and receipt of CAD software outputs from software developers. It is possible that the CAD software developers who received DICOM files from FIT had their own radiologists rapidly grade the test library so they could use their radiologist’s interpretations to influence or adjust their CAD software outputs before providing them to FIT. However, this likelihood was deemed to be low, particularly for commercially available CAD solutions, and recent CAD software evaluations have used similar methods for data collection^27,29^. To highlight the differences in data collection methods, and higher levels of trust in the CAD software outputs directly collected by FIT, all analyses in this manuscript have been presented by data collection method.

Despite these limitations, this independent evaluation has conclusively shown that TB program implementers now have a wide, and expanding, selection of accurate CAD software platforms to choose from when designing their programs. Comprehensive prospective operational evaluations are urgently needed to understand the optimal placement of CAD software in TB screening programs. Achieving the potential of CAD software to improve TB detection and treatment rates will depend on the availability of investments to scale-up implementation and ensuring optimal value and placement within the TB diagnosis and care cascade.

## Methods

### Mobile CXR screening

FIT has been organizing mobile CXR screening events for TB in Ho Chi Minh City, Viet Nam since 2017^9,33^. Community members who are at higher risk for TB (e.g. TB contacts, the elderly, people who have TB symptoms, people living far from health facilities, etc.) are mobilized in collaboration with district and commune health authorities and local partners^45^. Participants are first screened for TB symptoms (e.g. cough, fever, night sweats, weight loss, etc.) and other risk factors (history of TB, diabetes, HIV) using a questionnaire on a custom-built mHealth application. They are then referred for CXR screening regardless of their symptom presentation. CXR images are immediately read by a field radiologist and graded as normal or abnormal using the principle of ‘intentional over-reading’ in line with TB prevalence survey methods^46^. Individuals with an abnormal CXR result are asked to provide a good-quality sputum specimen at the screening event. At the end of each day, specimens are transported to a government laboratory for testing with the Xpert MTB/RIF assay (Cepheid, United States of America [USA]).

### Test library creation

**Figure 2:**
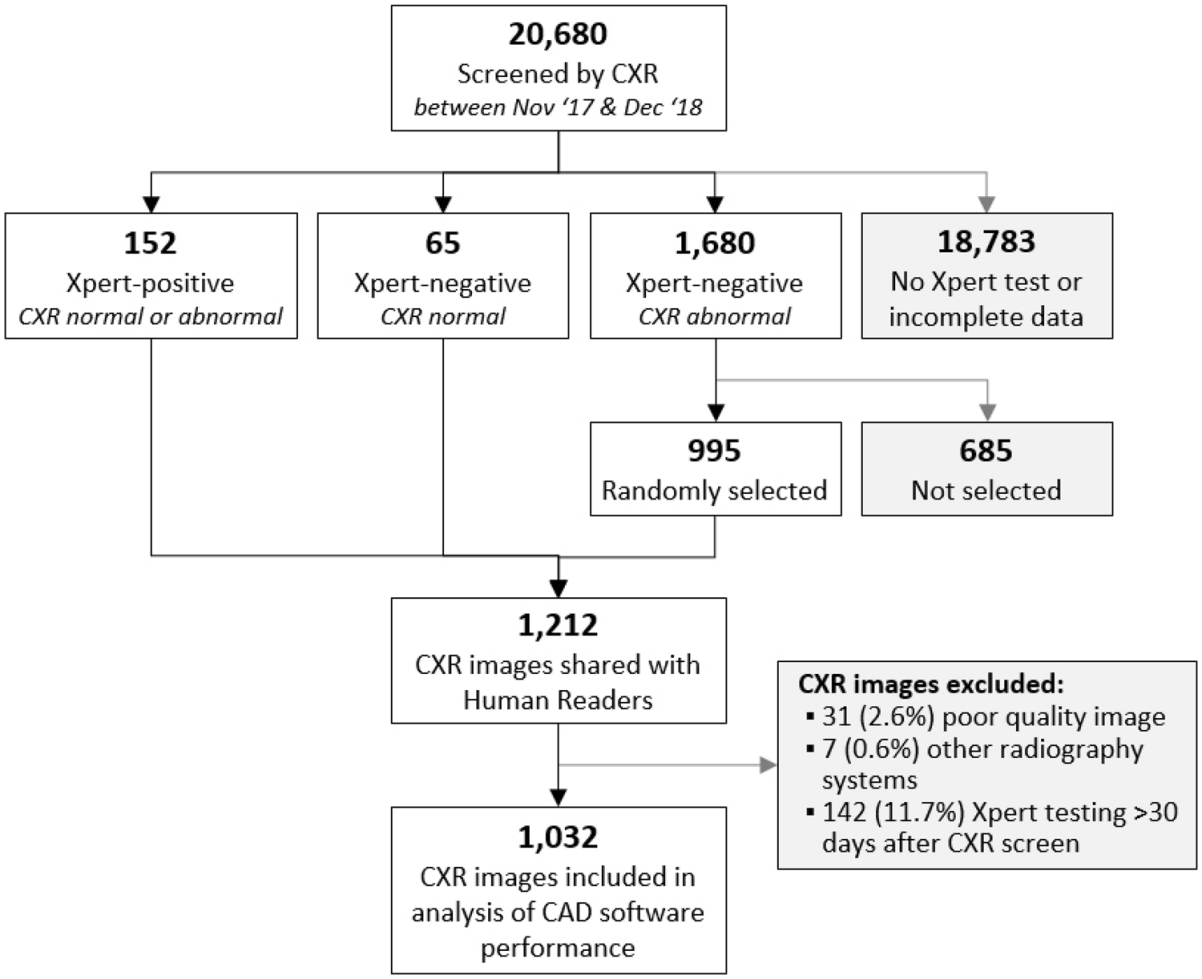
Diagram of test library creation.

DICOM files and clinical data from the mobile CXR screening events organized between 01 November 2017 and 31 December 2018 were used to create a purposively sampled test library for use in CAD software evaluations. The CXR images from 20,680 participants screened during this time period were reviewed for inclusion in the test library (Fig. 2); participants who did not have a valid Xpert test result (mostly because of an initial normal CXR result from the field radiologist), those who were aged less than 15 years, and/or individuals with foreign objects (e.g. pacemakers, jewelry, underwire, etc.) obscuring their lung fields were excluded. Three types of participants were ultimately selected: (1) all participants (n = 152) with a positive Xpert result regardless of their CXR result from the field radiologist, (2) all participants (n = 65) with a valid Xpert result after a normal CXR result from the field radiologist (off-algorithm testing), and (3) a randomly selected sample of 60% of the participants (n = 995) with negative Xpert results after an abnormal CXR result from the field radiologist. A test library of 1212 DICOM files was constituted using these initial inclusion criteria. The participant’s meta-data inside the DICOM files (e.g. name, birth year and age) were then anonymized.

The test library was sent to two TB clinicians who regularly read CXR images for their respective facilities, for blinded re-reading; the only participant information available to the re-readers was study ID. All CXR images were graded using a standardized interpretation definitions^46^. The Expert Reader had over 30 years of experience working at the Provincial Lung Hospital in Ho Chi Minh City, whereas the Intermediate Reader had 5 years of experience working at the Provincial Lung Hospital in Quang Nam, a lower TB burden province in the center of Viet Nam.

The test library was further refined after the blinded re-reads were obtained. Thirty-one CXR images which were graded as poor quality by either the Expert or Intermediate Readers were excluded. A total of seven different radiography systems were used during FIT’s mobile CXR screening events; however, just two radiography systems were used for 99% of the CXR screens. Thus, we excluded the seven CXR images which were captured by the other five radiography systems. Finally, 142 participants who were tested on Xpert more than 30 days after their CXR screen were also excluded. The final test library contains 1032 well-characterized CXR images (Fig. 2).

### CAD processing

Sixteen companies offering CAD software for TB screening were identified after a review of the literature and searches on the internet (Artelus, USA; Delft Imaging, The Netherlands; COTO, USA; DeepTek, India; Dr CADx, Zimbabwe; EPCON, Belgium; InferVision, Japan; JF Healthcare, China; JLK, South Korea; Lunit, South Korea; OXIPIT, Lithuania; Quibim, Spain; Qure.ai, India; RadiSen, South Korea; SemanticMD, USA; and Zebra Medical Vision, Israel). 14 companies signed collaboration agreements with FIT which outlined data sharing and the scope of the evaluation (all but Quibim and Zebra Medical Vision). Two companies later withdrew (JLK and RadiSen), leaving 12 companies included in the final evaluation report. Five of the CAD solutions included in this evaluation (DeepTek, CAD4TB, Lunit, Oxipit and Qure.ai) have obtained CE certification to date^47^.

DeepTek, Delft Imaging and Qure.ai provided FIT with direct access to their software through either an online user interface or an offline box system. The test library was processed and software outputs were collected directly by FIT staff for these three CAD companies. The test library was shared with all remaining CAD companies via a download link. Staff at these companies processed the DICOM files and provided their software’s outputs to FIT within 1 week of data sharing. De-identified demographic and clinical data, including CXR re-reads and Xpert results, were shared with all 12 CAD companies after their software outputs were obtained so these data could be used to train their software algorithms.

### Statistical analyses

Descriptive statistics summarizing participant demographics and clinical data were prepared, stratified by Xpert test result, and chi-squared tests were used to measure differences in Xpert positivity. The human reader CXR interpretations were recoded into a binary abnormal/normal result. Abnormal CXR images contained opacities/cavitation/lesions which were possibly caused by TB. CXR images containing abnormalities which the human readers were certain were of non-tubercular origin (e.g., canon ball metastases, vascular abnormalities, emphysema, etc.) were grouped with normal CXR images in this recorded variable. The analysis of CAD software outputs was disaggregated into two groups: abnormality scores obtained directly by FIT and scored provided by the CAD software developers. We first assessed the performance of each CAD software using their continuous abnormality score output. Receiver operating characteristic (ROC) curves were plotted using Xpert test results as the reference standard and areas under the curve (ROC AUCs) were calculated. In addition, we calculated the area under the precision-recall curve (PR AUC), due to the test library’s low overall Xpert positivity rate^48^. We then identified two cut-off thresholds to transform the continuous abnormality score of each CAD software into dichotomous normal/abnormal interpretations that matched the sensitivity achieved by the Expert and Intermediate Readers. Performance characteristics of each CAD software were then calculated at these two cut-off thresholds to allow for direct comparisons with human readers (primary outcome metric). For the seven CAD software solutions which performed at least on par with the Intermediate Reader, we calculated and quantitatively compared ROC AUCs^49^ across key demographic and clinical factors, including gender, age group, symptom status, history of TB and radiography system. Statistical analyses were performed using Stata version 13 (StataCorp, USA) and graphs were generated using R version 4.0.0 (R Foundation for Statistical Computing, Austria).

### Ethical considerations

Ethical approvals were granted by the Pham Ngoc Thach Hospital Institutional Review Board (430/HDDD-PNT) and the Liverpool School of Tropical Medicine Research Ethics Committee (17-019). Study implementation was approved by the Ho Chi Minh City People’s Committee (214/QD-UBND, 2138/QD-UBND). All participants provided written informed consent, and all methods were carried out in accordance with relevant guidelines and regulations. No patient-identifiable data were shared with CAD companies or were used for statistical analyses.
